# Branched Peptide, B2088, Disrupts the Supramolecular Organization of Lipopolysaccharides and Sensitizes the Gram-negative Bacteria

**DOI:** 10.1038/srep25905

**Published:** 2016-05-13

**Authors:** Rajamani Lakshminarayanan, Wei Xiang Tan, Thet Tun Aung, Eunice Tze Leng Goh, Nandhakumar Muruganantham, Jianguo Li, Jamie Ya Ting Chang, Neha Dikshit, Padmanabhan Saraswathi, Rayne Rui Lim, Tse Siang Kang, Vanniarajan Balamuralidhar, Bindu Sukumaran, Chandra S. Verma, Jayaraman Sivaraman, Shyam Sunder Chaurasia, Shouping Liu, Roger W. Beuerman

**Affiliations:** 1 Singapore Eye Research Institute, Ocular Chemistry and Anti-Infectives, Singapore; 2 Duke-NUS Graduate Medical School, Ophthalmology and Visual Sciences Academic Clinical Program, Singapore; 3 National University of Singapore, Department of Pharmacy, Singapore; 4 Duke-NUS Graduate Medical School, Program in Emerging Infectious Diseases, Singapore; 5 Ocular Immunology and Angiogenesis Lab, Department of Veterinary Medicine & Surgery, University of Missouri, Columbia, USA; 6 Agency for Science, Technology and Research (A^*^STAR), Bioinformatics Institute, Singapore; 7 National University of Singapore, Department of Biological Sciences, Singapore; 8 Nanyang Technological University, School of Biological Sciences, Singapore

## Abstract

Dissecting the complexities of branched peptide-lipopolysaccharides (LPS) interactions provide rationale for the development of non-cytotoxic antibiotic adjuvants. Using various biophysical methods, we show that the branched peptide, B2088, binds to lipid A and disrupts the supramolecular organization of LPS. The disruption of outer membrane in an intact bacterium was demonstrated by fluorescence spectroscopy and checkerboard assays, the latter confirming strong to moderate synergism between B2088 and various classes of antibiotics. The potency of synergistic combinations of B2088 and antibiotics was further established by time-kill kinetics, mammalian cell culture infections model and *in vivo* model of bacterial keratitis. Importantly, B2088 did not show any cytotoxicity to corneal epithelial cells for at least 96 h continuous exposure or hemolytic activity even at 20 mg/ml. Peptide congeners containing norvaline, phenylalanine and tyrosine (instead of valine in B2088) displayed better synergism compared to other substitutions. We propose that high affinity and subsequent disruption of the supramolecular assembly of LPS by the branched peptides are vital for the development of non-cytotoxic antibiotic adjuvants that can enhance the accessibility of conventional antibiotics to the intracellular targets, decrease the antibiotic consumption and holds promise in averting antibiotic resistance.

The worldwide increase in the emergence of multi-drug resistant and pan-drug resistant bacteria is posing a significant public health crisis, threatening the benefits achieved with current antibiotics^1^,^2^. The increasing trend in the antibiotic resistance among Gram-negative pathogens is worrisome as there has been limited number of new antimicrobial agents in development for targeting this group of pathogens^3^,^4^,^5^. Unfortunately, there has been a marked decline in the discovery and the development of new antibiotics due to unfavourable economic factors and regulatory challenges in gaining approvals^6^. With no new drugs in the pipeline, the apocalyptic scenarios surrounding drug-resistant bacteria are rapidly assuming an alarming reality^3^,^7^,^8^.

Gram-negative bacteria are characterized by the presence of an additional outer membrane (OM) in their cells which confers protection without compromising the transport of materials/nutrients essential for cell survival and is also regarded as its first line of defense against toxic agents^9^. OM is composed of an asymmetrical bilayer of polyanionic glycolipid lipopolysaccharides (LPS) on the outer leaflet and phospholipids on the inner leaflet. The organization of LPS in the outer leaflet is stabilized by the divalent cations and the role of LPS is to maintain the structural integrity while establishing the permeability barrier for the antibiotics^10^. The bilayered structure anchors several channel forming proteins which accomplish important functions such as solute and protein translocation and signal transduction^11^. The OM components impose restrictions on the physico-chemical properties of antibiotics that can access to the intracellular targets. Hydrophilic compounds (average clogD_7.4_ = −2.8 where D is sum of concentrations of ionized and unionized forms of the drugs in each of the two immiscible phases at pH 7.4) with molecular mass of 600 Da enter through water-filled channels of porins whereas aminoglycosides, macrolides, antimicrobial peptides and rifamycins enter through interactions with the outer membrane^12^,^13^. Consequently, there are 21 new antibiotics in clinical development for treating Gram-positive bacterial infections whereas no new classes of the US FDA approved antibacterial drugs to treat Gram-negative infections^14^,^15^.

Indiscriminate use of antibiotics in the last few decades has resulted in lack of efficacy and evolution of antimicrobial resistance. In the era of growing antibiotic resistance and increased mortality caused by drug-resistant pathogens, alternative strategies are needed. The combined use of antibiotic adjuvants i.e., compounds that can enhance the antimicrobial activity of conventional antibiotics, is a major way forward for combating antimicrobial resistance and extend the life span of current antibiotics^16^. It has been reported that compounds that chelate or displace the divalent cations present in the OM of Gram-negative bacteria could render Gram-negative bacteria susceptible to hydrophobic antibiotics^17^,^18^,^19^,^20^. However, the available permeabilizers lack bacterial specificity, alter the lipid metabolism of the mammalian cells and are cytotoxic at the effective concentrations^16^.

Disruption or destabilization of the supramolecular assembly of the OM in Gram-negative bacteria is an alternative method that may facilitate rapid uptake of antibiotics into the intracellular targets. However, the destabilizers must have high affinity for the OM components, viz. the LPS and should not interact with the antibiotics so that bacteriostatic and bactericidal activity could be achieved at the sub-lethal concentrations. We have previously shown that a di-branched peptide B2088 has substantial OM permeability, forms a tight complex with the LPS and excellent antibacterial activity against various Gram-negative pathogens^21^.

For simplicity we represent the sequence of the peptide as (RGRKVVRR)_2_*K*K, where *K* indicates branched lysine residue. Extending this work, we now investigate the mechanism of B2088-LPS interactions and examined its ability to sensitize multi-drug resistant Gram-negative bacteria to various classes of antibiotics. Finally, we determine the criteria for LPS disruption and OM permeability by replacing the two valine residues with natural/synthetic amino acid residues having alkyl or aromatic side chains. The high water/buffer solubility combined with non-cytotoxicity of these peptides unveil a new class of permeabilizers and sets the basis for the rational design of permeabilizers that could act synergistically with other antibiotics and may help overcome antibiotic resistance mechanisms.

## Results

### Biophysical characterization of LPS-B2088 interactions

**Figure 1:**
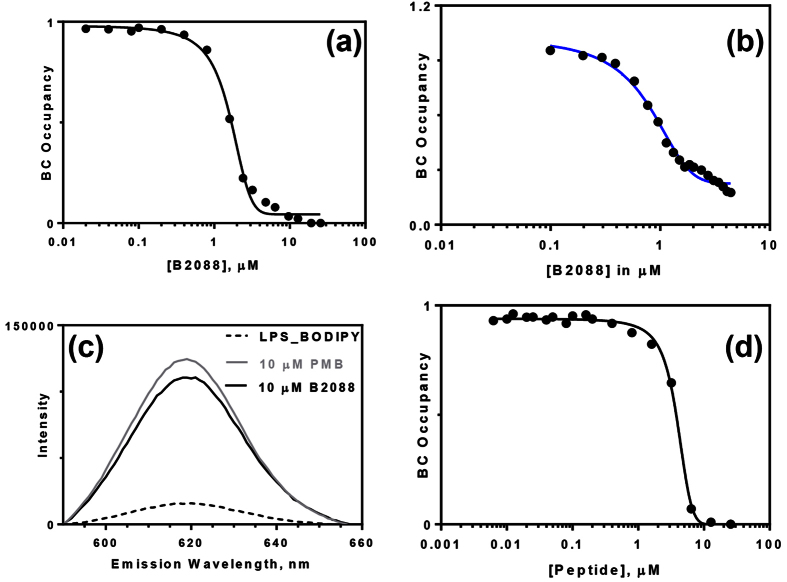
Interaction between LPS and B2088 probed by BC displacement assay. (**a**) Displacement of LPS-bound BC by the increasing concentration of B2088. (**b**) Displacement for lipid A-bound BC by B2088. (**c**) Fluorescence scans of the LPS-bound BC by polymyxin B (PMB) and B2088. The dotted lines represent the fluorescence spectra of LPS-bound BC. (**d**) Displacement of LPS-bound BC by linear retrodimer peptide.

To investigate the interaction of B2088 with LPS, we performed the BODIPY TR cadaverine (BC) displacement assay reported by Wood *et al*.^22^. The probe binds to the lipid A part of the LPS with moderate affinity and can be displaced by putative LPS-binding molecules. The method was used to probe the affinity of polycationic molecules and LPS binding proteins^23^,^24^. First, we determined the affinity of polymyxin B (PMB), a lipopeptide cyclic antibiotic which has a high affinity for LPS. An ED_50_ of 1.56 ± 0.02 μM (Supplementary Fig. S1) was observed for PMB which is in good agreement with a value of 2.5 ± 0.1 μM reported by Torrent *et al*.^24^. Figure 1a,b show the concentration-dependent displacement of LPS/lipid A-bound BC upon addition of B2088. The determined ED_50_ values for B2088 with LPS and lipid A were 1.92 ± 0.03 μM and 0.7 ± 0.14 μM, respectively, indicating that the peptide affinity for LPS/lipid A was comparable to PMB^25^. At an equivalent concentration, addition of PMB to LPS-BC complex showed a slightly higher fluorescence intensity compared to B2088 (Fig. 1c). To confirm the importance of branching, we determined the BC_50_ for a linear retrodimer having the amino acid sequence, RGRKVVRRKKRRVVKRGR (Fig. 1d). The determined value was 2-fold higher (3.9 ± 0.1 μM) than that of B2088, confirming a weaker affinity of the linear peptide.

### Interaction of B2088 with LPS is concentration-dependent and biphasic

**Figure 2:**
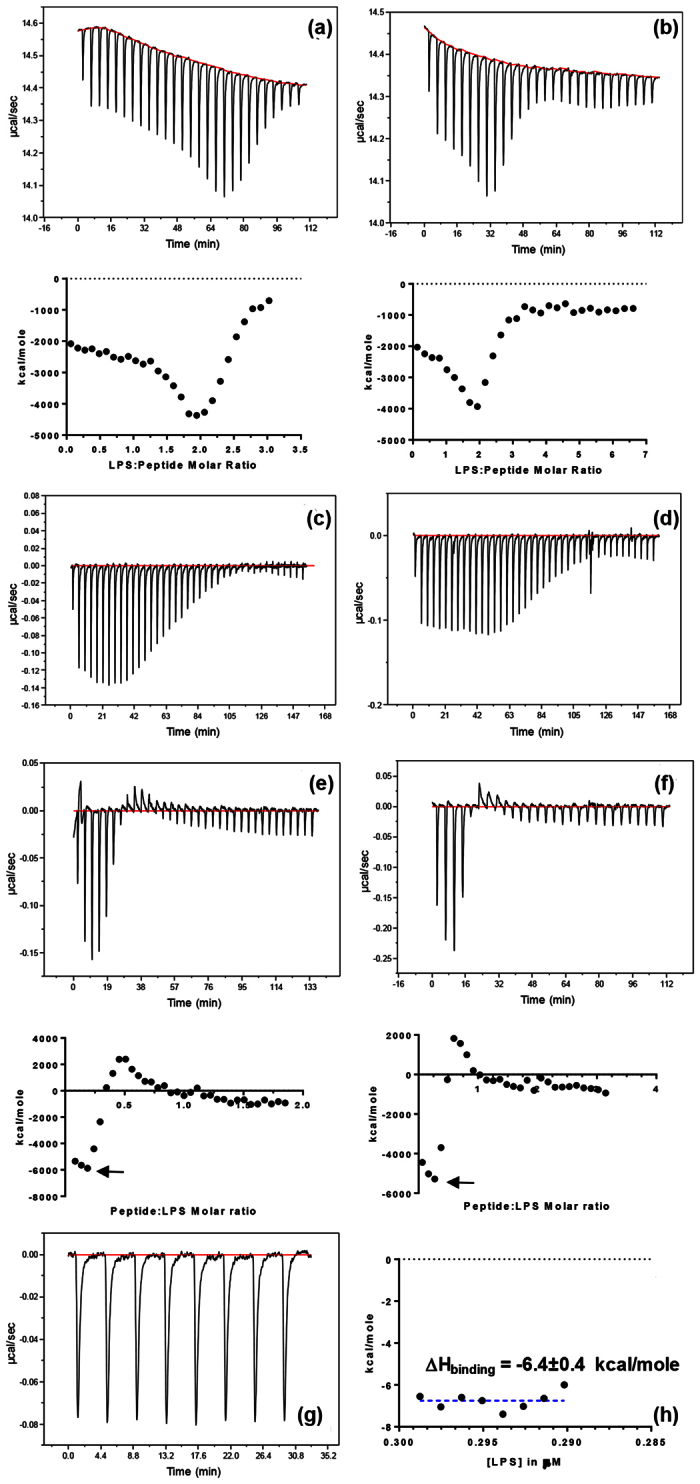
Dissecting the energetics of B2088-LPS interactions by ITC. (**a**) ITC traces showing the heat changes upon addition of 10 μL aliquots of LPS (315 μM) to 20 μM of B2088 in the sample cell. The bottom figure shows the enthalpy calculated for each titrations. (**b**) ITC traces showing the heat changes upon addition of 10 μL aliquots of LPS (240 μM) to 8 μM of B2088 in the sample cell. The bottom figure shows the enthalpy calculated for each titration. Note that LPS disruption to binding occurred earlier in B due to high initial LPS:peptide ratio. (**c**) ITC titrations showing addition of 4 μL aliquots of LPS (315 μM) to 10 μM peptide in the cell at 25 °C. (**d**) ITC titrations showing addition of 4 μL aliquots of LPS (315 μM) to 10 μM peptide in the cell at 37 °C. (**e**) ITC traces showing the heat changes upon addition of 5 μL aliquots of 150 μM B2088 to LPS (10 μM) in the sample cell. (**f**) The same experiment as in (**e**) but the injection volume was increased to 10 μL. Note that as the volume of injectant was increased a faster transition to LPS disruption (endothermic heat changes) occurred. (**g**) Steady-state ITC showing the heat changes upon addition of 6 μL aliquots of B2088 (60 μM) to a high concentration of LPS (300 μM) in the sample cell so that only binding reaction dominates. (**h**) Enthalpy change during steady-state ITC data and the blue dotted line indicates average enthalpy of binding. Note that the amount of LPS in the sample cell almost unchanged so that the total enthalpy is dominated by binding alone.

We used ITC to obtain better insights into the interaction of the peptide with LPS. When 10 μL of LPS solution was titrated into B2088 in the sample cell, a complex isotherm was observed (Fig. 2a). During the few initial injections, the exothermic heat remained unchanged, but began to increase as more LPS was added to the sample cell reaching a minimum. Further addition of LPS resulted in a decrease of the exothermic heat and reached a plateau as all the LPS had been added from the syringe. Decreasing the concentration of the peptide or LPS did not alter the shape of the titration curves (Fig. 2b). Similarly, decreasing the titrant volume (i.e., volume of LPS added in each injection) or temperature did not alter the shape of ITC traces as well (Fig. 2c,d). Titrating the peptide into the LPS in the sample cell (reversing the protocol) produced a trace that was almost a mirror image of the trace observed in Fig. 2a–d i.e., initial addition of LPS to peptide produced an exothermic heat changes which decreased with increasing concentration of the peptide (Fig. 2e,f). Further addition of peptide caused an endothermic heat changes, leading to a complicated ITC pattern. Integration of heat traces from Fig. 2a,b resulted in an ‘inverse bell’ shaped curve, suggesting a complex interaction between B2088 and LPS that may involve more than just binding interactions.

To dissect the energetics, we performed steady state ITC measurements wherein a large excess LPS was taken in the sample cell and an aliquot of peptide was titrated so that the enthalpy change is caused by one reaction^26^. Figure 2g shows the titration of 6 μL of B2088 into the sample cell containing a large excess of LPS, so that the reaction enthalpy is dominated by binding of the peptide to the LPS (ΔH_binding_). From the integration of the heat traces, an average enthalpy value of −6.8 ± 0.4 kcal/mole was obtained at 25 °C (Fig. 2h). It should be noted that, a similar value was obtained when the peptide was titrated into the LPS in the sample cell (indicated by black arrows in Fig. 2e,f). Subtraction of the binding enthalpy (ΔH_binding_) from the initial values obtained during the titration of LPS to B2088 (Fig. 2a–c), yields a value of +4.1 kcal/mole. These results suggest that titration of LPS to the peptide during the early stages may involve two competing reactions of opposite signs. During initial injection of LPS into a high concentration of peptide in the sample cell, peptide binding is coupled with dissociation of the LPS supramolecular structure. The dissociated monomeric form of LPS re-organizes and forms micellar or aggregated structures, contributing to endothermic enthalpy. Once the peptide concentration starts decreasing in the sample cell, further addition of LPS is dominated by binding enthalpy only, which then begins to decrease further until all the peptide is consumed. As a result, peptide-LPS interactions appear as a superimposition of two sigmoidal transitions leading to an “inverse bell”-shaped titration curve. Therefore, titration of LPS into the peptide is complicated by binding and dissociation enthalpy during the early stages (i.e., at low LPS:peptide ratio) whereas binding alone contributes to the enthalpy change at later stages (high LPS:peptide ratio). For simplicity we represent this enthalpy as ΔH_dis_. It should be noted that ΔH_dis_ is in fact associated with the formation of micellar aggregates from the monomeric LPS molecules that were formed during peptide-induced disruption of the LPS supramolecular structure. Thus we determined that, for B2088-LPS interactions, ΔH_binding_ contributes −6.4 kcal/mole whereas ΔH_dis_ contributes + 4.1 kcal/mole to the total enthalpies.

### Effect of B2088 on the supramolecular assembly of LPS

**Figure 3:**
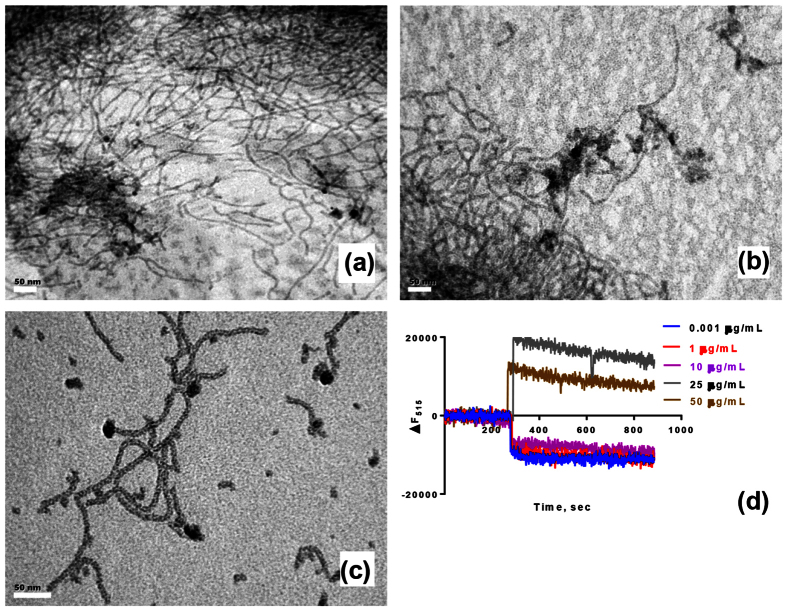
Characterization of LPS-B2088 interactions. (**a**) TEM images showing ribbon-like aggregates formed by LPS (**b**) Morphological changes in the LPS structure at B2088:LPS mole ratio of 0.04 and (**c**) Morphological changes in the LPS structure at B2088:LPS mole ratio of 2.5. Scale bar = 50 nm. (**d**) Changes in the fluorescence intensity of FITC-labelled LPS upon addition of B2088.

To confirm the findings from ITC experiments, we examined the ultrastructure of LPS or the LPS/B2088 complex by electron microscopy. TEM analysis of the LPS indicated a ribbon-like structure (Fig. 3a), consistent with previous studies^21^. For the LPS-B2088 complex, the peptide:LPS ratios were chosen to epitomize the initial (high peptide:LPS) or final (low peptide:LPS) injections of the ITC curves shown in Fig. 2a–c. At low peptide:LPS ratio (~0.04), more electron-dense surface appeared presumably due to binding of the peptide to LPS may cause partial/complete neutralization of the negative charges and the ribbon-like structure of LPS remained intact (Fig. 3b). However, when peptide:LPS ratio was increased (~2.5), considerable condensation in the length of ribbon-like structure was accompanied with formation of smaller LPS aggregates confirming that the peptide dissociates supramolecular organization of LPS (Fig. 3c). To further confirm these results, we probed the interaction between B2088 with FITC-labelled LPS (FITC-LPS) by monitoring the changes in the fluorescence intensity (Fig. 3d). The results suggested that the interaction is complicated by a biphasic transition: at low concentration of the peptide quenching of the FITC-LPS fluorescence intensity was observed whereas dequenching occurred above 25 μg/ml of the peptide. The dequenching of fluorescence intensity at higher peptide:LPS ratio suggests disassociation of the LPS supramolecular assembly^27^.

### B2088 binds to lipid A proximal region of the LPS

**Figure 4:**
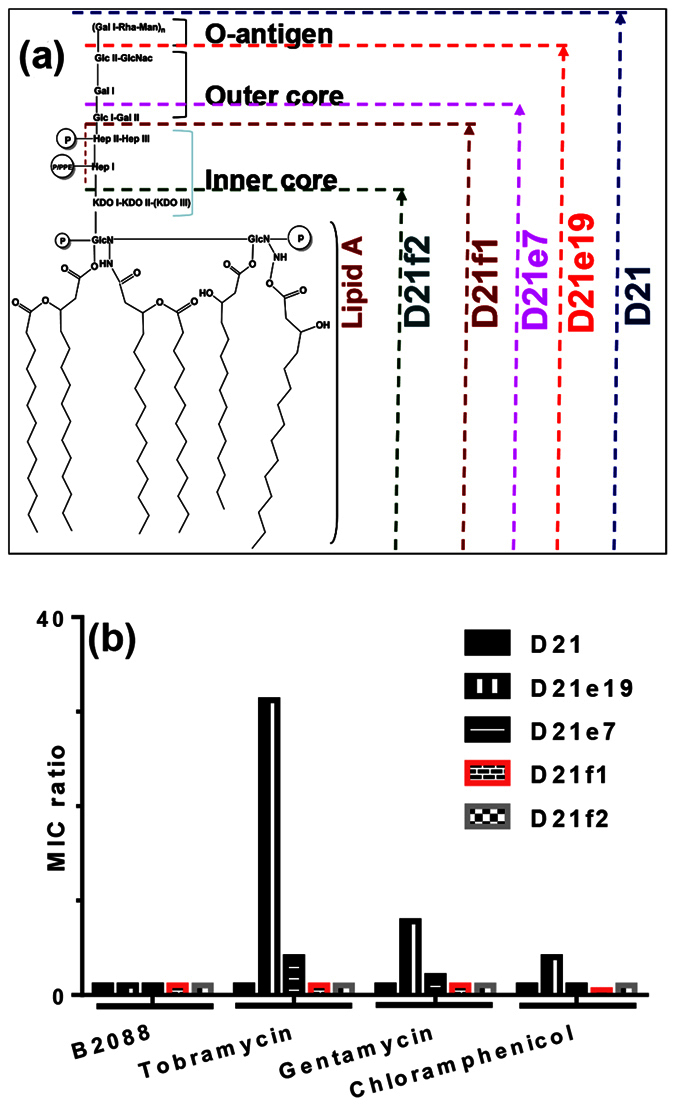
Effect of LPS truncation on antimicrobial activity of B2088 and antibiotics. (**a**) Schematic diagram showing the structure of LPS and the various truncated mutants. (**b**) MIC ratio for B2088 and antibiotics against various LPS-truncated mutants.

LPS is a complex glycosylated lipid molecule comprising of a hydrophobic lipid A and core oligosaccharides^28^. The core oligosaccharides are structurally divided into an inner core (lipid A proximal) region and an outer core O-antigen region (Fig. 4a). To ascertain if the alterations in OM polysaccharides modify the barrier penetration and antimicrobial properties of the peptides, we determined the MIC of the peptides against 4 cell-wall defective mutants of *E. coli* (Fig. 4a). We report the MIC ratio (i.e., MIC_mut_/MIC_wt_) which is the ratio of MIC of peptides/antibiotics against oligosaccharides truncated mutant strains over MIC of wild type (D21) strains. For antibiotics that access intracellular targets through porin pathways or peptides that bind to the lipid A part of the LPS, the MIC ratio is independent of LPS truncation (Fig. 4b). However, antibiotics or peptides which interact with outer or inner core polysaccharides of the LPS are expected to show changes in the MIC ratio. Carbinecillin and PMB (data not shown for clarity) did not show any change in the MIC ratio. However, a marked increase in the MIC ratio was observed for gentamicin, tobramycin and chloramphenicol against the mutant strains which lack outer core polysaccharides (D21e9). These results indicate that interaction of aminoglycosides with O-antigen is important for their antibacterial activities. As observed for PMB, no apparent change in the MIC ratio was observed for B2088, confirming that the peptide may bind to the lipid A part of the LPS.

### Molecular Dynamics Simulations of B2088-lipid A interactions

**Figure 5:**
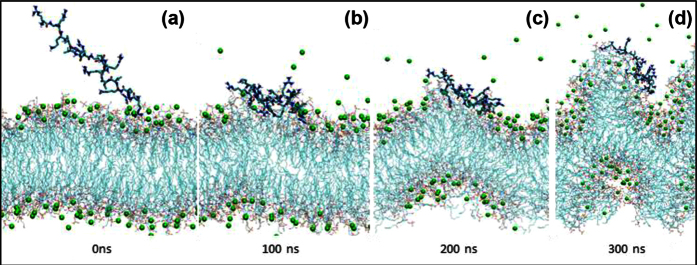
Molecular Dynamic (MD) simulations of peptide-lipid A interactions. Snapshots of B2088 interactions with a model lipid A bilayer from MD simulations. (**a**) 0 ns, (**b**) 100 ns, (**c**) 200 ns and (**d**) 300 ns. The peptide is represented in sticks, the calcium ions are in green spheres while the lipid molecules are denoted by lines.

To obtain atomistic insights underpinning the binding of B2088 to lipid A, we performed atomistic MD simulations of B2088 with a model lipid A membrane. Before adsorption of B2088, the lipid A membrane was stabilized by the ionic interactions between phosphate groups and divalent ions such as Ca^2+^. During the simulation, B2088 adsorbed rapidly (within 50 ns) onto the surface of lipid A (Fig. 5a,b), driven by electrostatic interactions between the negative charged head groups of lipid A molecule and the cationic residues of B2088. Once fully adsorbed onto the lipid A membrane, the side chains of cationic residues of B2088 such as Arg and Lys began to form hydrogen bonds with the phosphate groups of lipid A head groups (Fig. 5c,d). In particular, Arg side chain forms multi-dentate hydrogen bonds with the phosphate groups, consistent with previous observations^29^,^30^. As the simulation time progressed, the calcium-PO_4_^2−^ salt bridges were destabilized by the formation of intense hydrogen bonds between phosphate head groups and cationic residues with concomitant displacement of calcium ions into the aqueous solution. The interaction of B2088 also caused considerable charge imbalance across the two sides of the membrane. As a result, the lipid A membrane began to deform and undergoes significant perturbations after 300 ns. However, even after 400 ns, the linear retrodimer caused weak perturbation of the bilayered membrane (Supplementary Fig. S2). These results indicate that the multidentate interactions caused displacement of divalent ions and the concomitant perturbation of lipid A may contribute to the dissociation of LPS assembly.

### B2088 sensitizes *P. aeruginosa* to antibiotics *in vitro*

Based on the above observations, we inferred that B2088 binds to the lipid A part of the LPS with moderate affinity and disrupts the supramolecular organization of the LPS, thus may sensitize pathogenic bacteria to other antibiotics. To test this hypothesis we assessed the synergism between B2088 and various classes of antibiotics against a multidrug resistant *P. aeruginosa 4877* strains. As shown in Table 1, depending on the type of antibiotics, B2088 decreased the MIC values by 2-8 fold at sub-lethal concentrations of the peptide. In particular, antibiotics that gain access to the intracellular targets by outer membrane pathways displayed good synergism with the peptide. For example, the presence of sub-inhibitory concentrations of the peptide resulted in a 4-fold decrease in the MIC of chloramphenicol, tobramycin, kanamycin and gentamicin; for other antibiotics (except ciprofloxacin), only a moderate decrease in the MIC was observed. It should be noted that the fractional inhibitory concentration index (FICI) is ≤1 for 11 antibiotics and no antagonistic effect was observed with any of the tested antibiotics. Polymyxin B, however, displayed weaker synergistic effects with various classes of antibiotics when compared to B2088-antibiotics combinations against the same strains (Supplementary Table S1). In addition, for the linear retrodimer, an additive effect (FICI = 1) was observed for gatifloxacin, chloramphenicol and tobramycin antibiotics, indicating the importance of branching in sensitizing the bacteria. Synergism between B2088 and selected antibiotics was also investigated against *Klebsiella pneumoniae* strains, after determining the MIC of individual peptide/antibiotics (Supplementary Table S2). When tested against 3 different strains of *K. pneumoniae*, similar FICI values were observed for B2088 with chloramphenicol, tobramycin, gentamicin and gatifloxacin against two strains (Table 2). These results suggest the ability of B2088 to sensitize different strains of *P. aeruginosa* and *K. pneumoniae*.

**Figure 6:**
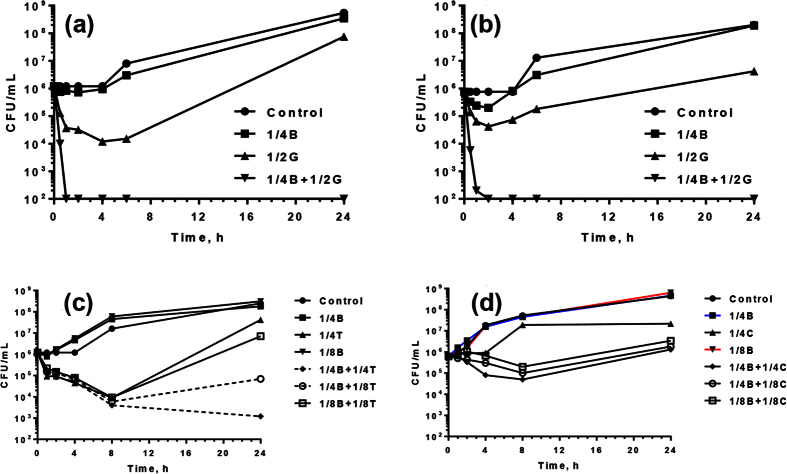
Antimicrobial efficacy of B2088-antibiotics combinations. Time-kill kinetics showing synergism between B2088 and other antibiotics. (**a**) B2088 and gatifloxacin against *P. aeruginosa* ATCC 9027 strains. (**b**) B2088 and gatifloxacin against *P. aeruginosa* DM4877/07 strains. (**c**) B2088 and tobramycin against *P. aeruginosa* DM4877/07 strains. (**d**) B2088 and chloramphenicol against *P. aeruginosa* DM4877/07 strains. Note that for simplicity B2088 and the antibiotics are represented by single letter codes (B – B2088, G – gatifloxacin, T – tobramycin, C – chloramphenicol).

It is likely that disruption of the supramolecular organization of LPS could enhance the intracellular accessibility of the antibiotics thus rendering the micro-organisms susceptible at sub-lethal concentration of the drugs. To confirm this, we performed time-kill kinetics at sub-lethal concentration of B2088, antibiotics and their combinations against two different *P. aeruginosa* strains. At ¼ × MIC of B2088 no bacteriostatic or bactericidal activity was observed, as shown by the increase in bacterial CFU/ml with time (Fig. 6a–d). At sub-MIC of antibiotics, after an initial decrease during the early stages, the bacterial populations grew with time and reached a maximum after 24 h (Fig. 6a–d). However, significant lethality was observed at sub-MIC combinations of B2088 and gatifloxacin/tobramycin as >5 log_10_ reduction in CFU/ml could be achieved when compared to an initial inoculum (Fig. 6a–c). The effect was quite remarkable for the B2088-gatifloxacin combination as >5 log_10_ reduction in CFU/ml was apparent in less than 2 h against two different *P. aeruginosa* strains. A similar lethal effect was observed at ¼ × MIC of B2088-tobramycin combinations. For B2088-chloramphenicol combinations at their ¼ × or ⅛ × MIC, a complete inhibitory effect was observed after an initial decrease during the early stages. These results support the checkerboard and biophysical studies that disruption of the supramolecular assembly of LPS may enhance the faster uptake of antibiotics, thus leading to rapid bactericidal activity.

### B2088-gatifloxacin/tobramycin combinations decrease intracellular bacterial load

**Figure 7:**
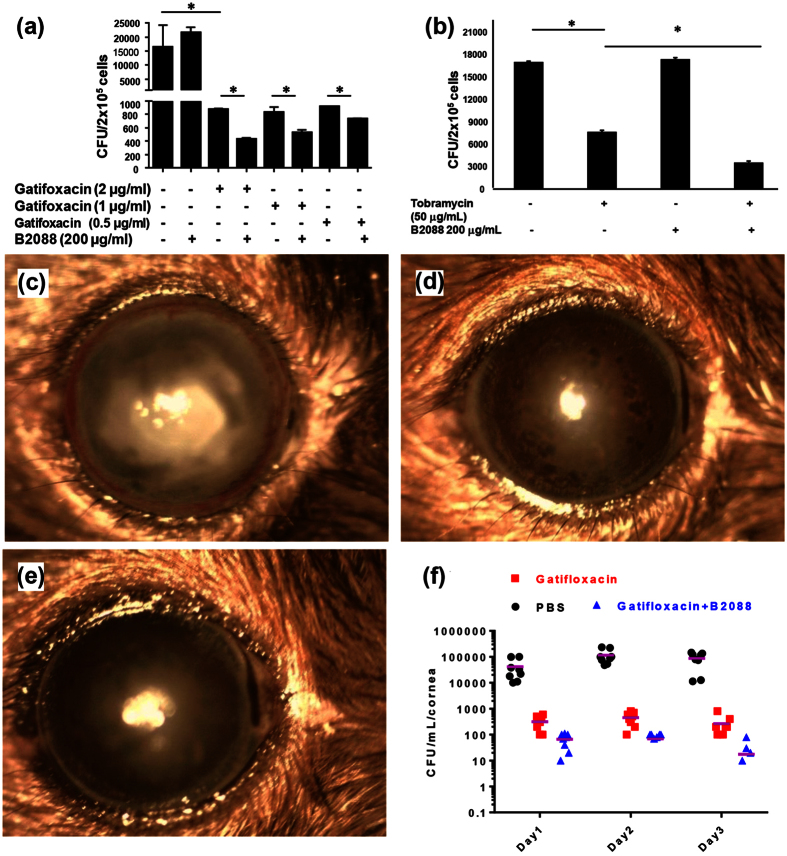
Efficacy of B2088-gatifloxacin combination in mammalian cell culture and mice model of bacterial keratitis. Decreased intracellular bacterial load by B2088 in combination with (**a**) gatifloxacin and (**b**) Tobramycin in infectedA549 cells. *Represents p < 0.05. Slit lamp biomicrocopy of mice treated with (**c**) PBS, (**d**) 0.3% gatifloxacin and (**e**) B2088 (0.05%) and 0.15% gatifloxacin after 3 days post treatment. (**f**) Bacterial burden in the untreated and treated cornea on days 1, 2 and 3 post infection. The pink bar indicates the average value.

*P. aeruginosa* has been shown to invade the corneal epithelial cells during infection and the invaded pathogen evades antibiotic therapy. To ascertain if the peptide and the drug combinations decreased the intracellular bacterial loading, we used A549 cells infected with *P. aeruginosa* ATCC 9027 strains. After 24 h post infection, gatifloxacin/tobramycin treatment decreased the intracellular bacterial load (Fig. 7a). B2088 (at 200 μg/ml) treatment alone did not decrease the intracellular bacterial burden. At 2 μg/ml, gatifloxacin showed marked decrease in the intracellular bacterial burden. However, treatment with B2088 along with gatifloxacin resulted in a significant decrease in the intracellular bacterial load when compared to antibiotics alone or in untreated cells. Interestingly, B2088 in combination with 0.5 μg/ml of gatifloxacin produced similar results as that of cells treated with 2 μg/ml of gatifloxacin alone. A similar decrease in the bacterial burden was observed in the presence of tobramycin-B2088 combinations, thus confirming the synergistic efficacy of peptide-antibacterial combinations in decreasing the intracellular bacterial burden (Fig. 7b).

### *In vivo* efficacy of B2088-gatifloxacin combination therapy

The marked synergism between B2088 and gatifloxacin in the mammalian cell culture model prompted us to investigate the efficacy of the combinations *in vivo*. A mouse model of keratitis was used to examine the efficacy of peptide-antibiotics combinations. After infection of the cornea with *P. aeruginosa ATCC 9027* strains, mice were treated with gatifloxacin (0.15% w/v) or B2088 (0.05%) alone as well as gatifloxacin (0.15%) in combination with B2088 (0.05% w/v). Infected mice mock-treated with PBS served as control, which displayed a severe clinical course and increased bacterial burden 3 days post infection. (Fig. 7c,d and Supplementary Fig. S3). Mice treated with topical application of 0.3% gatifloxacin displayed clear cornea and less bacterial load compared to PBS-treated mice (Fig. 7e). Mice cornea treated with 0.15% gatifloxacin in combination with 0.05% B2088 showed a lower bacterial burden compared to mice treated with 0.3% gatifloxacin (Fig. 7f). We have observed an increased bacterial burden in the cornea of mice treated with 0.15% gatifloxacin or 0.05% B2088 alone (Supplementary Fig. S3), indicating that the presence of B2088 decreased the bacterial burden and reduced the antibiotic use by about 50%, thus establishing the synergism with gatifloxacin *in vivo*.

### B2088 is non-cytotoxic and non-haemolytic to mammalian cells

**Figure 8:**
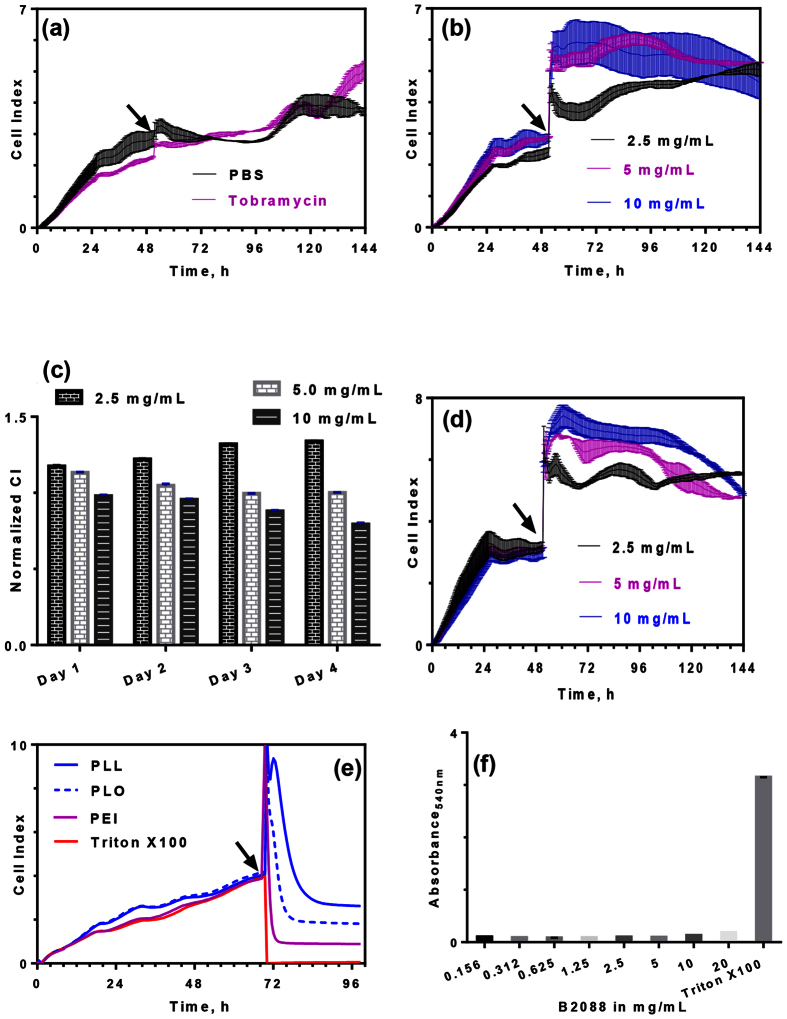
Evaluation of cytotoxicity of B2088 to mammalian cells. (**a**) Dynamic monitoring of cell viability expressed as cell index (CI) by xCELLigence system. hTCepi (15,000 cells/well) cells were allowed to attach to the electrode surface and exposed to PBS or 2.5 mg/ml tobramycin (indicated by black arrow). (**b**) Effect of B2088 on cell index. The time of addition is indicated by black arrow. (**c**) Normalized CI values determined after the addition of B2088 at various time intervals. (**d**) Effect of cell density (30,000 cells/well) on cytotoxicity of B2088. (**e**) Cytotoxicity of cationic OM permeabilizers to hTCEpi cells at 125 μg/ml. The abbreviations used are: PLL – poly(L-lysine); PLO - poly(L-ornithine); PEI - linear polyethylenimine. For a comparison the sensogram for the cytotoxic surfactant Triton-X100 is also shown. For better clarity only average value is depicted in the figure without standard deviation. (**f**) Hemolytic activity of B2088 to rabbit erythrocytes.

To assess the safety of B2088 as a part of preclinical assessment, we used the xCELLigence system for the real-time analysis of cytotoxicity. The method monitors the electronic impedance of adhering cells in terms of cell index (CI) and does not require any labelling molecules. Figure 8a shows a typical CI signal for a buffer or non-toxic drug (tobramycin). For peptide B2088, no adverse decrease in the CI values were observed even at 10 mg/ml up to 4 days, confirming lack of any cytotoxic effect of the peptide to the human corneal epithelial cells (Fig. 8b). Interestingly, a significant increase in CI values (p < 0.005) was observed with time at low concentration of B2088, indicating the promotion of cell proliferation (Fig. 8c). In addition, no cytotoxicity was observed with increased cell density (Fig. 8d). On the other hand, polycationic agents (poly-L-lysine, poly-L-ornithine or polyethyleneimine) that were reported to sensitize OM of Gram-negative bacteria displayed marked cytotoxic effect at 125 μg/ml (Fig. 8e). Rabbit erythrocytes exposed to 20 mg/ml of B2088 did not show any hemolysis thus confirming high specificity of B2088 in discriminating between prokaryotic and eukaryotic cells (Fig. 8f).

### Effect of hydrophobic amino acid residues on antimicrobial activity and OM permeability

To obtain more insights into the structure-function relationship of interactions between B2088 and LPS, we modified the two hydrophobic valine residues in each copies of the peptide by various proteinogenic or non-proteinogenic amino acid residues. Table 3 compares the antimicrobial properties of various peptide congeners modified from B2088. Substitution by alanine or norvaline caused 2–8 fold increase in the MIC values, suggesting the importance of hydrophobicity and isopropyl side chain of valine residues. However, such effect was not obvious in the case of leucine, isoleucine or norleucine substitution, as all the three congeners displayed similar MIC values. The effect of bulky amino acid residues was clearly visible when the two valine residues in B2088 were substituted with bulky aromatic amino acid residues. Substitution with phenyl alanine resulted in moderate increase in the MIC values whereas peptides such as biphenyl alanine or tryptophan showed higher MIC values.

Next, we examined the LPS binding, OM permeability and synergism with other antibiotics for the various peptide congeners (Supplementary Figs S4 and S5). A clear trend was apparent among amino acid residues having aliphatic and aromatic side chains (Table 4). Substitution with aliphatic residues did not alter the ED_50_ and PC_50_ values significantly, although the alanine substituted congener displayed the weakest OM permeability.

Consistent with the MIC results, phenyl alanine substituted congener displayed the strongest affinity and permeability amongst all other peptides and residues having bulky substituents showed only moderate affinity and permeability. Synergism studies showed that peptide congeners having norvaline, phenylalanine and tyrosine substitutions displayed the best synergism with aminoglycosides and chloramphenicol. In contrast, bulky aromatic substituents caused weak synergism or additive effect with the antibiotics. It is interesting to note that none of the peptides displayed any antagonistic effect with the tested antibiotics, suggesting that the peptides do not interact with antibiotics or interfere with their uptake pathways.

## Discussion

Excessive or widespread use of antibiotics in healthcare and environment related industries has dramatically increased the frequency of antibiotic resistance among human pathogens. Bottlenecks in developing new antibiotics pipelines, unfavourable economic profits and stringent regulatory norms complicate the problem further, leading to an “antibiotic-apocalypse”-like situation^31^,^32^,^33^. Decreasing the permeability (influx) as well as increasing the active efflux of antibiotics are the hallmarks of multidrug-resistant phenotypes^34^,^35^. In particular, modification of LPS, porin loss, reduced expression of porins and modification of the porin channels can decrease the antibiotic susceptibility to hydrophilic antibiotics^36^,^37^. Therefore, chemical entities that could enhance or facilitate antibiotic uptake by interfering with the microbial protection mechanisms is a major step forward in combating antimicrobial resistance. A number of cationic agents and ion chelators have been shown to sensitize Gram-negative bacteria to hydrophobic antibiotics. These molecules bind to the divalent cations or displace them from the intact packing of LPS, thus weakening the OM structure^20^,^38^,^39^,^40^,^41^,^42^,^43^,^44^.

The critical micellar concentration of LPS is very low (sub-picomolar) and forms a supramolecular assembly of ribbon-like structure at nM concentrations, thus making biochemical characterization challenging^45^. Using BC fluorescence dequenching experiments we showed that B2088 binds to LPS with almost equal affinity as PMB. Based on ITC experiments, we conclude that the energetics of interaction between LPS and B2088 is a complex process and involves two stages: a strong electrostatic interaction between the cationic peptide and the anionic LPS (binding) at lower concentration and disruption of supramolecular assembly of LPS at higher peptide concentration which is accompanied by the association of the disrupted LPS monomers to form large aggregates. The association of monomer LPS into aggregates involves considerable removal of water molecules from hydrophobic tails, thus characterized by endothermic heat changes^46^. As a result, the total enthalpy at high peptide:LPS ratio is contributed by competing binding and disruption-followed by micellization (of LPS) processes that results in the anomalous patterns in the ITC curves. Such complex behavior in the ITC experiment has also been reported by others for LPS/lipid A interactions with polymyxin B or polymyxicin B nonapeptide, N-lauryl lactoferrin derived peptide (lauryl-LF11), cationic antimicrobial peptides or polymers^47^,^48^,^49^,^50^. However, none of these reports accounted for the observed biphasic transitions in these cases.

The concentration-dependent biphasic transitions observed in the fluorescence studies (FITC-labelled LPS and B2088) and disruption of the ribbon-like structures and formation of LPS aggregates (by TEM) at high peptide:LPS ratio support the results derived from ITC analysis. Similar increase in fluorescence intensity upon LPS disruption was observed for cationic and amphipathic antimicrobial peptides^27^,^51^.

Recently, Malojicic *et al*., have shown concentration-dependent changes in the interaction between LPS transport protein E (LptE) and LPS by surface plasmon resonance and electron microscopy^52^. These authors further demonstrated that key amino acid residue substitutions in LptE resulted in defective OM biogenesis and rendered Gram-negative bacteria susceptible to rifampin and vancomycin, which are otherwise inactive antibiotics against these pathogens. Similarly, Warren’s group demonstrated marked synergism between collistin and glycopeptide antibiotics (vancomycin and Teicoplanin) against *A. baumannii* strains, suggesting that disruption of OM barriers may expand the antibiotics armamentarium against multidrug-resistant Gram-negative bacteria^53^,^54^,^55^.

Using various LPS truncated mutants that are deficient in outer core polysaccharides, we showed that B2088 binds to the lipid A part of the complex LPS structure. Consistent with these results, MD simulations results suggest that the binding affinity of B2088 arises mainly from (i) high charge density and (ii) multi-dentate hydrogen bonding of B2088 with the phosphate head groups of lipid A, which resulted in considerable disruption of the lipid A assembly and displacement of the divalent cations.

The moderate binding affinity of peptide for LPS and the peptide’s ability to modify the aggregation status of LPS prompted us to investigate if B2088 could sensitize a multi-drug resistant *P. aeruginosa* against various classes of antibiotics. Interestingly, the studies demonstrated that the presence of sub-lethal concentration of B2088, caused 2–8 fold decrease in the MIC of various classes of antibiotics. Low FICI values (≤0.5) were observed with aminoglycosides and chloramphenicol antibiotics which access their intracellular targets by outer membrane path ways^9^,^10^. In contrast, moderate to weak synergism (FICI > 0.5 to <1) was observed for antibiotics that access the intracellular targets by porin pathways. Consistent with these results, time-kill kinetics studies showed a marked decrease in growth inhibition or rapid bactericidal action with three different classes of antibiotics. The absence of any interaction between the peptide and other antibiotics and its ability to disrupt the LPS structure may have increased the antibiotics uptake, thus eliciting rapid bactericidal action at sub-lethal concentrations. Reproducibility of the results in a mammalian cell culture model and *in vivo* model of infectious keratitis further strengthen our argument that B2088 sensitizes *P. aeruginsoa* and enhances the antibiotics uptake. The presence of 0.05% peptide in combination gatifloxacin decreased the antibiotic use by about 50% with better *in vivo* activity compared to drug or peptide alone.

As a measure of mammalian cell biocompatibility, we evaluated the cytotoxicity of B2088 by label-free xCELLigence assay. The method is non-invasive and temporal changes can be monitored for an extended period of time continuously. The lack of any cytotoxic effect on epithelial cells with continuous exposure for four days and non-hemolytic activity even at high concentrations highlighted the safety and selectivity of the peptide. In contrast, cationic OM permeabilizers such as PLL, PLO or PEI, at their effective permeabilizing concentrations, displayed substantial cytotoxicity^38^,^56^.

Altering the hydrophobicity of the peptides with natural or unnatural amino acid residues containing alkyl or aromatic substitutions resulted in dramatic changes in antimicrobial and OM permeability. Substitution of valine with alanine caused substantial decrease in both the antimicrobial activity and synergism with other antibiotics whereas the norvaline congener showed better synergism despite a significant decrease in its antimicrobial activity. Of the 4 aromatic amino acid substituted congeners, peptides containing phenyl alanine showed the best synergistic activity with only a marginal decrease in antimicrobial activity. However, substitution of bulky aromatic amino acid residues resulted in considerable loss of antimicrobial activity, OM permeability and synergism with other antibiotics. These results are quite different from what has been observed for linear or cyclic peptides wherein tryptophan substitution caused enhanced OM permeability and synergism with other antibiotics^57^,^58^,^59^,^60^. Though very preliminary, these studies provide rationale for designing new generation of non-cytotoxic and water/buffer soluble branched peptides with enhanced OM permeability and synergism with conventional antibiotics.

In summary, using a multidisciplinary approach, we have unravelled the mechanism of LPS-B2088 interactions. The results derived from our work establish synergistic efficacy *in vitro* and *in vivo*, biocompatibility of the peptide and design of new peptides with improved *in vitro* efficacy. Further studies on the rational design of more efficient OM permeabilizing peptides and their ability to increase the uptake of various classes of antibiotics may not only expand the life span of existing antibiotics but also expand the antibiotic armamentarium against multi-drug resistant pathogens.

## Materials and Methods

All the peptide congeners were purchased from M/s. Mimotopes Pty Ltd, Australia. The peptides were used as such and the homogeneity of peptides were confirmed by HPLC. Unless otherwise stated all the chemicals and the antibiotics tested were purchased from Sigma Aldrich (Singapore) Pte Ltd. The florescent probe, BODIPY TR cadaverine (BC) was purchased from Molecular Probes/ Invitrogen (CA, USA). *E. coli* LPS outer core polysaccharide truncated mutant strains were obtained from CGSG, The Coli Genetic Stock Centre, Yale University, NH, USA. Relevant genotype of the strains are, D21 (rfa+); D21e19 (rfa-11) is a deficient mutant in carbohydrates close to the O-antigen part; D21e7 (rfa-1) is deficient in galactose as well as O-antigen; D21f1 (rfa-1, rfa21) lacks phosphate groups in the LPS has glucose and heptose intact; D21f2 (rfa-1, rfa-31) is deficient in heptose of the inner core^57^,^58^.

### Determination of the Minimum Inhibitory Concentration (MIC)

MIC of each peptide was determined using the microdilution method in a 96 well microtitre plates (SPL Life Sciences Co., Ltd, Korea). Two-fold serial dilutions of each peptide in Mueller-Hinton Broth (MHB) was prepared from a 1 mg/ml stock solution of the peptide in the same broth, to obtain a final concentration in the range of 0.0976–50 μg/ml in each well. Bacterial suspensions (*P. aeruginosa* ATCC 27853, *P. aeruginosa* 4877, *P. aeruginosa* 23155, *K. pneumoniae* ATCC 10031 and *K. pneumoniae* 4299) were prepared by suspending the overnight cultured bacteria in saline and adjusting the inoculum to ~10^8^ colony forming units (CFU) per ml. A further 150-fold dilution was then prepared in MHB to achieve a final cell density of 10^5^–10^6^ CFU/ml. In each well, 100 μl of inoculum was mixed with the equal volume of peptides at their 2× concentration. Positive and negative controls contained 200 μl of inoculum (without any peptide) and broth alone (no peptide/inoculum) respectively. Another negative control in which MHB containing 200 μl of peptide alone in its highest concentration tested (50 μg/ml) was also included to ensure that the optical density measured in the test wells was not due to turbidity of the peptides in solution. The 96 well polystyrene plates were then incubated at 37 °C for 24 h and the optical density at 600 nm (OD_600_) was measured every 30 min continuously using a TECAN microplate reader (TECAN Infinite 200, Austria). All the experiments were performed in two independent duplicates and the MIC was determined as the lowest peptide concentration in which no visible growth was observed.

### Checkerboard Assay

The synergism between peptides with conventional antibiotics was evaluated using checkerboard assays. For a comparison polymyxin B (PMB) was also tested against each antibiotic. From their stock solutions, two-fold dilutions of each antibiotic and peptide to 1× MIC were prepared in MHB. 50 μl of each peptide concentration was added into the wells of a 96- well polystyrene plates (SPL Life Sciences Co. Ltd, Korea) in a horizontal orientation and the same volume of each antibiotic concentration were then added in a vertical orientation. Bacterial suspensions were prepared in MHB to yield final cell density of ~10^6^ CFU/ml and 100 μl of bacterial suspension was mixed with each peptide-antibiotic combinations. This procedure was repeated for wells containing 200 μl of peptide alone and 200 μl of antibiotic alone in various concentrations. The positive and negative controls contained 200 μl of inoculum and MHB alone respectively. The plates were incubated at 37 °C for 24 hours and the optical density at 600 nm (OD_600_) was monitored as before. Fractional inhibitory concentration index (FICI) for all antibiotic- peptide combinations was calculated through the summation of (MIC of peptide in the combination/ MIC of peptide alone) and (MIC of antibiotics in combination/MIC of antibiotic alone). The experiments were performed in two independent duplicates and the combinations that gave the lowest FICI values was reported.

### Time-kill Kinetics Studies

The kinetics of bactericidal action of is assessed by using each drug alone or in combinations at their sub-inhibitory MICs. The time-kill studies were performed with a final inoculum of 10^5^–10^6^ CFU/ml in MHB with the peptides/antibiotics at ½, ¼ and **⅛ × **of their MICs. The tubes were incubated at 37 °C under continuous agitation. Duplicate samples are withdrawn at various time intervals (0–24 h) and the log_10_-fold dilutions were plated onto a tryptic soy agar (TSA) plate for CFU determination.

### Transmission electron microscopy (TEM)

TEM images of LPS or LPS-peptide complex were acquired with a JEOL JEM-1010 transmission electron microscope using Digital Micrograph™ (Gatan, Pleasanton, CA) available at the National University of Singapore Electron Microscopy Unit facility. Formvar-carbon coated 300-mesh-size nickel grids were placed in an invert position on top of an aliquots of 10 μl solution from LPS/LPS-peptide for 30 minutes. Excess samples were blotted off the filter paper and negatively stained with 10% phosphotungstic acid (PTA). The grids were dried and observed imaged by electron microscope at 80 kV.

### FITC-LPS interactions

The interaction between FITC-LPS was determined by monitoring the change in fluorescence emission intensity (λ_ex_ = 485 nm and λ_em_ = 515 nm) using a Quanta Master spectrophotometer (Photon Technology International, NJ, USA) with a constant slit width of 0.5 nm at 25 °C. Briefly, the emission intensity of 5 μg/ml of FITC-LPS was monitored continuously in a 1 ml cuvette until it remained constant. 10 μl a concentrated stock solution of B2088 was added and the changes in fluorescence intensity were recorded as before.

### Isothermal Titration Calorimetry

ITC was performed by using a VP-ITC microcalorimeter (Microcal, MA, USA). The peptide and LPS solution in phosphate buffer (pH = 7.0) were degassed under vacuum and conditioned (by titrating water-to-water injection) before each titration so as to ensure no traces from previous runs. In the first set of experiments, small aliquots (4–10 μL) of LPS (310 μM) was injected into the sample cell (V_cell_ = 1.43 ml) containing the peptide (10–15 μM). In a separate experiment, the peptide was taken in the syringe and titrated into the LPS solution taken in the cell. For steady state ITC experiments, peptide in the syringe was titrated against a large excess of LPS in the cell. For all these experiments the temperature was maintained at 25 ± 0.3 °C or 37 ± 0.4 °C.

### BODIPY TR Cadevarine (BC) Displacement Assay

The BC displacement assay was used to determine the ability of each peptide to bind to LPS. The florescent probe, BODIPY TR cadaverine (BC) was purchased from Molecular Probes/ Invitrogen (USA). A stock solution containing 1 mg/ml of LPS (*E. coli* serotype 055:B5) was prepared in dimethyl sulfoxide (DMSO) and another stock solution containing 1 mg/ml of BC dye was prepared in 5 mM HEPES buffer (pH 7.4). The optimum saturation concentration of BC:LPS ratio was standardized following the protocol provided by Wood *et al*. (22). From the BC stock, 240 μl was mixed with 500 μl from the LPS stock solution in 5 mM HEPES buffer (pH 7.4) to prepare 20 ml of BC:LPS mixture. Two-fold serial dilutions of each peptide in 5 mM HEPES buffer (pH 7.4) was prepared from their 1 mg/ml stock solutions to obtain a final range of 0.02–25.6 μM. The BC:LPS mixture was added to 100 μl of each peptide congener in a 1:1 ratio in each well of a 96-well black Costar™ polystyrene microtitre plate (Corning Incorporated, NY, USA). The maximum fluorescence intensity (F_max_) was determined using a mixture containing 100 μl PMB (50 μg/ml) and 100 μl BC:LPS solution, as PMB is considered the ‘gold standard’ for LPS-sequestering agents. The minimum fluorescence intensity (F_0_) was measured using 200 μl of BC:LPS mixture to determine the background noise for this experiment. Fluorescence measurements for each peptide (F) were determined using a microplate reader (Model: TECAN Infinite 200, Austria) with excitation and emission wavelengths set at 580 nm and 620 nm respectively. The percentage of BC displacement was then calculated using the formula:


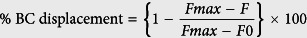


The experiments were performed in duplicates and the effective concentration required for 50% displacement of BC dye (ED_50_) from LPS with respect to PMB was determined by curve fitting using Graphpad Prism (CA, USA). A higher ED_50_ value for the peptide indicates a lower affinity for LPS.

### Outer Membrane Permeability Assay

The ability of each peptide to permeabilize the OM of Gram-negative organisms was determined using the hydrophobic fluorescent probe 1-*N*-phenylnaphthylamine (NPN) as previously reported^21^. *P. aeruginosa* ATCC 9027 cells from an overnight MHB culture were harvested after centrifuging at 3000 rpm for 10 minutes. The pellet was washed twice, suspended in 5 mM HEPES (*N*- 2-hydroxyethylpiperazine-*N*’-2 ethanesulfonic acid) buffer (pH 7.4) and adjusted to an OD_600_ of 0.200. A suspension containing 600 μl of bacteria cells and 1 μl of a concentrated stock solution of NPN was mixed into a 10 mm stirred quartz cuvette so that the final concentration of the probe remained 10 μM. Aliquots containing 1 μl of each peptide in various concentrations (100, 50, 25, 15, 10, 5, 2.5 and 1 μg/ml) were added upon NPN dye stabilization. The fluorescence intensity was monitored using a Quanta Master spectrophotometer with a constant slit width of 0.5 nm, excitation and emission wavelengths set at 355 nm and 405 nm respectively. The peptide concentration required for half-maximal intensity (PC_50_) was determined by sigmoidal curve fitting using Graphpad Prism software. The experimental data is average of two independent titration experiments.

### Antimicrobial activity in A549 cell culture model

Human lung A549 epithelial cells (ATCC CCL185) were seeded at a density of 10^5^ cells/well in a 24 well plate. Next day cells were infected with *P. aeroginosa* 9027 strains at the multiplicity of infection of 5 for 2 h. Cells were then washed thrice with PBS followed by DMEM to remove the extracellular bacteria. The washed cells were subsequently treated with 200 μg/ml gentamicin to kill any extracellular bacteria. After 1 h cells were washed with PBS again to remove the dead bacteria and the cells were either mixed with antibiotics (Tobramycin or Gatifloxacin) alone or in combination with B2088. After 24 h, the cells were lysed with 0.1% Triton-X100 and the lysates were serially diluted and plated on to a tryptic soy agar (TSA) plates for colony counting.

### *In vivo* antimicrobial activity in a mice model of infectious keratitis

Wild type C57BL6 (6–8 weeks old and weighs about 20-30 gram) female mice purchased from National University of Singapore were used for this study. *P. aeruginosa* ATCC 9027 was grown overnight in TSA (Tryptic Soy Agar) at 37 °C. Concentration of 3 × 10^8^ bacterial stock was prepared for mice corneal infection with United States Phosphate Buffer (USP) by resuspending few colonies from the overnight cultured TSA plates.

Healthy mice with good corneal clarity were chosen for this study through careful monitoring with slit-lamp biomicroscopy. A total of 60 mice were used for this study (n = 12 for 5 groups) at different time points (day1, day2, day3 post treatment). The animals were anesthetized subcutaneously by 0.2 ml of ketamine (100 mg/ml) and 0.1 ml of xylazil (20 mg/ml) in 0.7 ml of normal saline (0.08 ml/mice). A sterile mini blade (BD Beaver, MA, USA) was used to de-epithelialize the corneal surface (each 1 mm long) of the right eye, which did not breach beyond the superficial stroma, whereas the left was untouched. Topical application of bacteria suspension (10 μl) was applied to the scratched cornea. All the animals had been treated with the antibiotics or peptide/antibiotics combinations (3 times/day) started from day1 post infection.

At post treatment day1, day 2 and day 3, four animals from each group were euthanized. The infected corneas was dissected and processed for colony counting. Single individual cornea from different groups was homogenized in sterile 0.9% NaCl containing 0.25% BSA and diluted serially (10^1^ to 10^2^ fold dilution), plated in duplicates on TSA plates and incubated for 48 h at 37 °C. The number of viable bacteria was enumerated as before.

All the animals used in this study were treated in accordance with the tenets of the Association for Research in Vision and Ophthalmology (ARVO) statement and the protocol was approved by Singhealth Institutional Animal Care and Use Committee (IACUC; AALAC accredited, #2013/SHS/827).

### Molecular Dynamic Simulations

Atomistic molecular dynamics simulations were carried out to study the mode of interactions of B2088/linear retrodimer with a model lipid A bilayer consists of 64 lipid A molecules and 64 calcium ions^61^. Gromos 53a6 force field was used to model both the peptide and the lipid molecules. Initially, the peptide was placed close to the lipid A bilayer. The peptide-membrane complex was solvated with SPC water and addition Ca^2+^ were added to neutralize the system. Before the production state of MD simulations, the system was first subjected to 500 steps of energy minimization using the steep descent algorithm, followed by 100 ps of NVT simulation. During the MD simulations, LJ and short-range electrostatic interactions were treated with cut-off scheme, while long range electrostatic interactions were calculated using PME method^25^. The simulation was run in NPT ensemble with temperature maintained at 300 K through the Nose-Hoover method and pressure maintained at 1 atm using the Parrinello-Rahman method with semi-isotropic method. The simulation was performed using GROMACS package 4.6^62^.

### Cytotoxicity of B2088 to corneal epithelial cells

The xCELLigence system (ACEA, CA, USA) was used to assess the cytotoxicity of B2088. Human telomerase immortalized corneal epithelial (hTCEpi) cells, a kind gift from Prof. Winston Kao (University of Cincinnati, Ohio), were cultured in serum-/ calcium-free keratinocyte culture media (DermaLife, Lifeline Cell Technology, MD, USA) supplemented with provided reagents and maintained at 37˚C in 5% CO_2_. Electronic impedance in the form of cell index (CI) was derived corresponding to the relative density and adherence strength of cells in each well. hTCEpi (15,000 or 30,000 cells per well) was seeded on an e-plate 96. Confluency of cells in each well was determined according to CI values. At 70–80% confluency, culture media was removed and replaced with hTCEpi media with indicated concentrations of peptides (2.5–10 mg/ml) of peptides in triplicates. Polymers (PLL, PLO and PEI) were added at a final concentration of 125 μg/ml. Tobramycin (2.5 mg/ml) and 10 mM PBS (pH 7.0) served as positive controls in the same e-plate whereas Triton X100 served as a negative control. Culture was maintained for 96 hours with no media change. CI values of treated groups were normalized once the values stabilized after the addition of peptide.

### Haemolytic activity of peptides

Serial two-fold dilutions of peptides in PBS was mixed with rabbit red blood cells (final concentration 4% v/v) and incubated at 37 °C for 1 h. The mixture was centrifuged at 3000 rpm for 10 min and the release of hemoglobin in the supernatant was monitored by measuring the hemoglobin absorbance at 540 nm. The readings from cell suspension in PBS (without any additives) or 1% Triton-X100 served as 0% or 100% hemolysis, respectively.

## Additional Information

**How to cite this article**: Lakshminarayanan, R. *et al*. Branched Peptide, B2088, Disrupts the Supramolecular Organization of Lipopolysaccharides and Sensitizes the Gram-negative Bacteria. *Sci. Rep.* **6**, 25905; doi: 10.1038/srep25905 (2016).

## Supplementary Material

Supplementary Information

## Figures and Tables

**Table 1:** Synergism between B2088 and various classes of antibiotics.

Antibiotics	MIC of antibiotics in μg/ml	MIC of antibiotics in μg/ml in the presence of indicated sub-MIC of B2088*			FICI†
		½×	¼×	⅛×	
Chloramphenicol	200	25	50	100	**0.5**
Tobramycin	800	200	200	–	**0.5**
Gentamicin¶	0.39	0.024	0.098	0.195	**0.5**
Kanamycin	3200	800	1600	–	0.75
Streptomycin	200	50	100	100	0.75
Nalidixic acid	3200	400	–	–	0.63
Ciprofloxacin	12.5	–	–	–	NS
Levofloxacin	50	12.5	–	–	0.75
Gatifloxacin	31.25	7.8	15.6	15.6	0.63
Imipenem	0.78	0.0975	0.195	0.5	**0.5**
Carbenicillin	1600	200	800	–	0.63
Erythromycin	100	50	–	–	1.0

Combinations that gave best synergism (FICI = ≤ 0.5) are shown in bold.

^*^For this work *P. aeruginosa DR4877/07* strains (from sputum) was used. MIC of B2088 against this strain was 6.25 μg/ml.

^¶^*P. aeruginosa DR4877/07* strain showed remarkable resistance to gentamicin that we could not determine the MIC. Therefore, FICI was determined against the *P. aeruginosa ATCC 9027* strains.

^†^B2088-antibiotics combinations that gave the lowest FICI values are shown in the table. ‘–’ indicates no complete inhibitory effect at the indicated sub-MIC concentrations.

**Table 2:** Synergism between B2088 and other antibiotics against 3 different *K. pneumoniae strains*.

Strains	Fractional Inhibitory Concentration Index with			
	Tobramycin	Gentamicin	Gatifloxacin	Chloramphenicol
*K. pneumonia 4299*	**0.25**	**0.37**	0.56	**0.38**
	(0.125,0.125)	(0.125,0.25)	(0.06,0.5)	(0.13,0.25)
*K. pneumonia 07955*	0.75	0.75	0.63	**0.38**
	(0.5,0.25)	(0.25,0.5)	(0.13,0.5)	(0.13,0.25)
*K. pneumonia 31158*	**0.25**	**0.31**	0.56	0.63
	(0.125,0.125)	(0.25,0.06)	(0.06,0.5)	(0.5,0.13)

Combinations that gave the best synergism (FICI = ≤ 0.5) are shown in bold. ^**†**^The numbers in parenthesis indicate the fractional inhibitory concentration of B2088 and antibiotics used for FICI calculations.

**Table 3:** Effect of hydrophobic amino acid residues substitution on the antimicrobial properties of B2088.

Peptides	Minimum Inhibitory Concentration (μM) against				
	*P. aeruginosa DR4877/07*	*P. aeruginosa ATCC 27853*	*P. aeruginosa 23155*	*K. pneumoniae ATCC10031*	*K. pneumonia 4299*
B2088 (RGRKVVRR)_*2*_*K*K	2.7	2.7	1.4	1.4	2.7
(RGRKAARR)_*2*_*K*K	11.5	11.5	11.5	11.5	23
(RGRKN_val_N_val_RR)_*2*_*K*K†	5.5	10.9	5.5	10.9	21.8
(RGRKLLRR)_*2*_*K*K	2.7	2.7	2.7	5.3	5.3
(RGRKN_leu_N_leu_RR)_*2*_*K*K†	2.7	2.7	2.7	2.7	5.3
(RGRKIIRR)_*2*_*K*K	5.3	2.7	5.3	2.7	5.3
(RGRKFFRR)_*2*_*K*K	5.0	5.0	5.0	5.0	10.1
(RGRKF_bi_F_bi_RR)_*2*_*K*K†	4.9	9.8	4.9	9.8	19.6
(RGRKYYRR)_*2*_*K*K	9.0	4.5	4.5	4.5	9.0
(RGRKWWRR)_*2*_*K*K	19.0	9.5	9.5	9.5	19
Polymyxin B	1.13	0.57	0.57	0.57	1.13

^†^N_val_ – norvaline, N_leu_ – nor leucine and F_bi_ – biphenyl alanine.

**Table 4:** LPS binding, OM permeability and synergism between various peptide congeners modified based on B2088 sequence and other antibiotics.

Peptides	BC_50_	PC_50_	Fractional Inhibitory Concentration Index with*				
			*Erythro*	*Levo*	*Tobra*	*Genta*†	*Chloarm*
**B2088**	1.9 ± 0.1	13.2 ± 0.4	1.0	1.0	**0.5**	**0.5**	**0.5**
			(0.5,0.5)‡	(0.5,0.5)	(0.25,0.25)	(0.25,0.25)	(0.25,0.25)
(RGRK**AA**RR)_***2***_***K***K	2.1 ± 0.2	21.7 ± 0.8	1.0	0.75	0.75	0.56	0.63
			(0.5,0.5)	(0.5,0.25)	(0.5,0.25)	(0.5,0.06)	(0.5,0.13)
(RGRK**N**_**val**_**N**_**val**_RR)_***2***_***K***K	1.45 ± 0.01	14.9 ± 1.2	1.0	0.75	**0.38**	**0.38**	**0.5**
			(0.5,0.5)	(0.5,0.25)	(0.25,0.13)	(0.25,0.13)	(0.25,0.25)
(RGRK**LL**RR)_***2***_***K***K	1.47 ± 0.02	17.5 ± 0.4	1.0	0.56	**0.5**	**0.5**	**0.38**
			(0.5,0.5)	(0.5,0.06)	(0.25,0.25)	(0.25,0.25)	(0.25,0.13)
(RGRK**N**_**leu**_**N**_**leu**_RR)_***2***_***K***K	1.62 ± 0.02	16.4 ± 0.1	0.53	0.75	0.56	0.51	0.56
			(0.5,0.03)	(0.5,0.25)	(0.06,0.5)	(0.5,0.01)	(0.5,0.06)
(RGRK**II**RR)_***2***_***K***K	1.44 ± 0.02	17.0 ± 0.2	1.0	0.63	0.56	0.51	0.75
			(0.5,0.5)	(0.5,0.13)	(0.5,0.06)	(0.5,0.01)	(0.5,0.25)
(RGRK**FF**RR)_***2***_***K***K	0.93 ± 0.01	10.0 ± 0.3	1.0	1.0	**0.256**	**0.5**	**0.38**
			(0.5,0.5)	(0.5,0.5)	(0.25,0.06)	(0.25,0.25)	(0.25,0.13)
(RGRK**F**_**bi**_**F**_**bi**_RR)_***2***_***K***K	2.33 ± 0.06	22.2 ± 1.6	1.0	1.0	**0.50**	0.56	0.56
			(0.5,0.5)	(0.5,0.5)	(0.25,0.25)	(0.5,0.06)	(0.5,0.06)
(RGRK**YY**RR)_***2***_***K***K	2.5 ± 0.04	21.9 ± 0.2	0.56	0.75	**0.38**	**0.5**	**0.38**
			(0.5,0.06)	(0.5,0.25)	(0.25,0.13)	(0.25,0.25)	(0.25,0.13)
(RGRK**WW**RR)_***2***_***K***K	6.8 ± 0.12	31.1 ± 4.6	1.0	0.75	0.75	0.56	0.63
			(0.5,0.5)	(0.5,0.25)	(0.5,0.25)	(0.5,0.06)	(0.5,0.13)
**Polymyxin B**	1.56 ± 0.02	5.82 ± 0.6	0.75	0.75	1.0	1.0	0.63
			(0.25,0.5)	(0.5,0.25)	(0.5,0.5)	(0.5,0.5)	(0.125,0.5)

Antibiotics with excellent synergism are highlighted in bold.

^*^For all the peptides-antibiotics combinations *P. aeruginosa DR4877/07* was used (except peptides-gentamicin combinations).

^†^For peptides-gentamicin combinations *P. aeruginosa ATCC 27853* strains was used. MIC of gentamicin against this strain was 0.04 μg/mL.

^‡^The numbers in parenthesis below the FICI values indicate the fractional inhibitory concentration of peptides/PMB and antibiotics used for the calculations.
