# Supplementary material for: Branched Peptide, B2088, Disrupts the Supramolecular Organization of Lipopolysaccharides and Sensitizes the Gram-negative Bacteria
Source: Sci Rep. 2016 May 13;6:25905. doi: 10.1038/srep25905 (PMC4865820; doi:10.1038/srep25905)
Supplement: Supplementary Information [file srep25905-s1.pdf]

Branched Peptide, B2088, Disrupts the Supramolecular Organization of Lipopolysaccharides and Sensitizes the Gram-negative Bacteria

Lakshminarayanan Rajamani[1,2,*], Wei Xiang Tan[3], Thet Tun Aung[1], Eunice Tze Leng Goh[1], Nandhakumar Muruganantham[1], Jianguo Li[1], Jamie Ya Ting Chang[1], Neha Dikshit[4], Padmanabhan Saraswathi[1], Rayne Rui Lim[1,5], Tse Siang Kang[3], Vanniarajan Balamuralidhar[4], Bindu Sukumaran[4], Chandra S Verma[6,7,8], Jayaraman Sivaraman[7], Shyam Sunder Chaurasia[1,5], Shouping Liu[1,2,*], and Roger W Beuerman[1,2,*]

[1]Singapore Eye Research Institute, Ocular Chemistry and Anti-Infectives, Singapore.

[2]Duke-NUS Graduate Medical School, Ophthalmology and Visual Sciences Academic Clinical Program, Singapore.

[3]National University of Singapore, Department of Pharmacy, Singapore.

[4]Duke-NUS Graduate Medical School, Program in Emerging Infectious Diseases, Singapore.

[5]Ocular Immunology and Angiogenesis Lab, Department of Veterinary Medicine & Surgery, University of Missouri, Columbia, USA.

[6]Agency for Science, Technology and Research (A*STAR), Bioinformatics Institute, Singapore.

[7]National University of Singapore, Department of Biological Sciences, Singapore.

[8]Nanyang Technological University, School of Biological Sciences, Singapore.

[*]Corresponding Authors

Dr. Rajamani Lakshminarayanan
Email: lakshminarayanan.rajamani@seri.com.sg

Dr. Shouping Liu
Email: liu.shou.ping@seri.com.sg

Prof. Roger Beuerman
Email: rwbeuerman@gmail.com

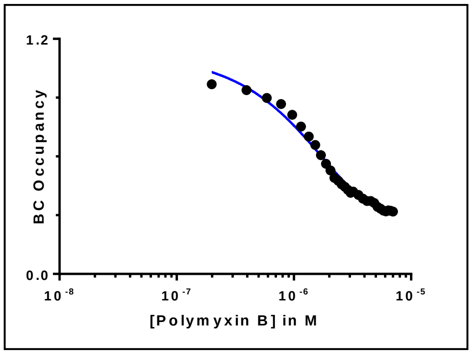

Supplementary Figure S1. Interaction of polymyxin B with LPS probed by BC displacement fluorescence assay.

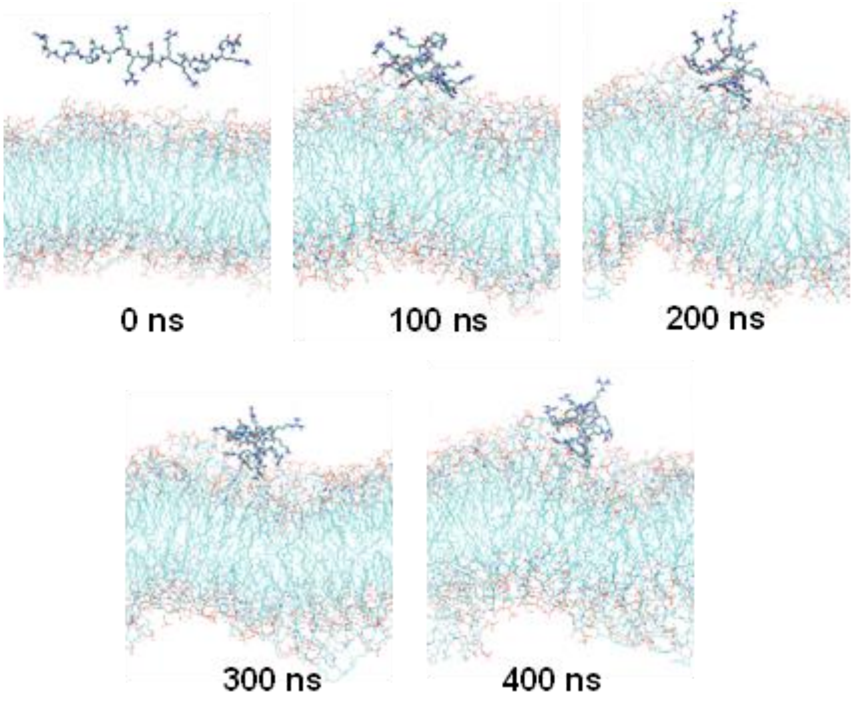

Supplementary Figure S2. MD simulations of linear retropeptide-lipid A interactions. Snapshots of B2088 interactions with a model lipid A bilayer from MD simulations at various time intervals. The peptide is represented in sticks, the calcium ions are in green spheres while the lipid molecules are denoted by lines. Note the absence of a clear deformation of the bilayer even longer time intervals.

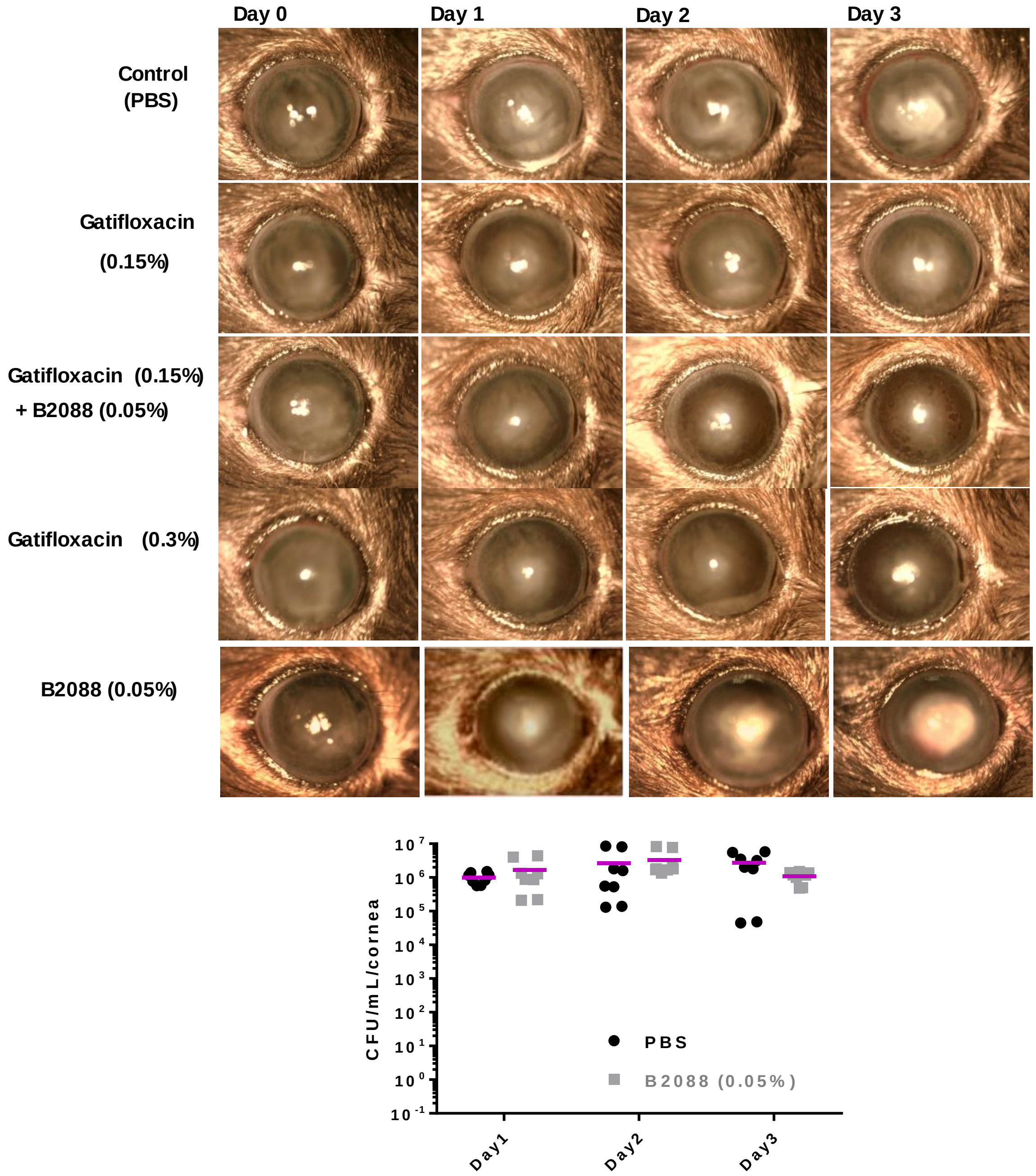

Supplemental Figure S3. (top) Slit lamp biomicroscopy images showing course of corneal infections in the untreated, gatifloxacin treated as well as gatifloxacin-peptide combinations treated cornea. (bottom) Bacterial burden in the cornea treated with PBS or B2088 (0.05% w/v) after 1-3 days post infection.

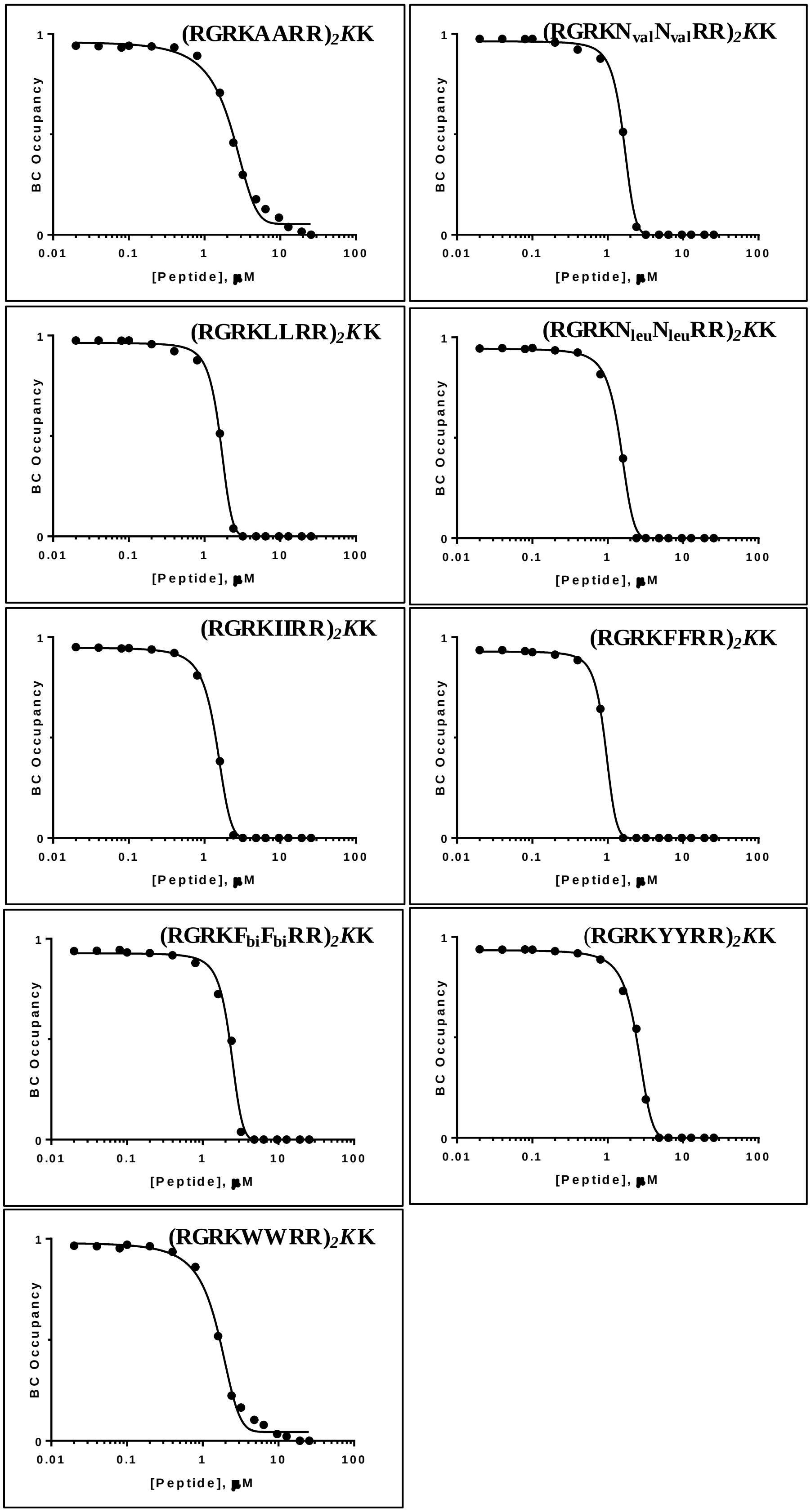

Supplemental Figure S4. Interaction of various peptide congeners with LPS probed by BC assay. The sequence of the peptide congeners are indicated in the figure.

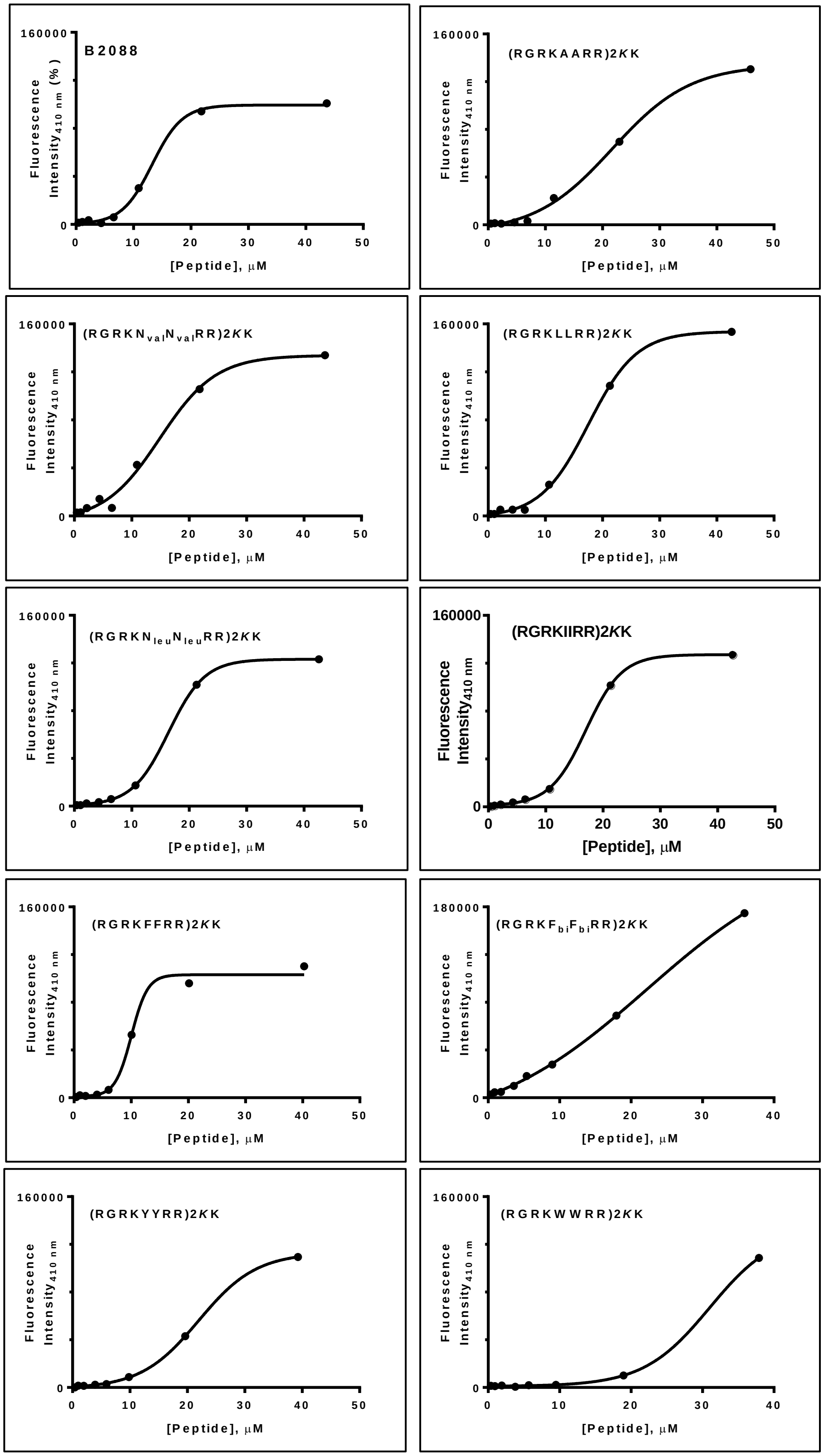

Supplemental Figure S5. OM permeability of various peptide congeners probed by NPN assay and determination of PC_{50}.

Table S1. Synergism between polymyxin B and various classes of antibiotics

Antibiotics	MIC of antibiotics in μg/ml	MIC of antibiotics in μg/ml in the presence of indicated sub-MIC of Polymyxin B			FICI
		½ ×	¼ ×	⅛ ×	
Chloramphenicol	200	50	50	50	0.63
Tobramycin	800	0.018	0.037	0.037	1.0
Gentamycin[¶]	0.39	0.195	-	-	1.0
Kanamycin	3200	200	1600	-	0.56
Streptomycin	200	100	100	-	0.5
Nalidyxic acid	3200	1600	-	-	1.0
Ciprofloxacin	12.5	12.5	12.5	-	0.75
Levofloxacin	50	12.5	-	-	0.75
Gatifloxacin	31.25	3.91	15.63	-	0.63
Imipenam	0.78	0.39	0.195	-	0.75
Carbenicillins	1600	1600	1600	1600	0.56
Erythromycin	100	50	50	-	0.75

[*]For this work *P.aeruginosa DR4877/07* strains (from sputum) was used. MIC of Polymyxin B against this strain was 1.56 μg/ml. [¶] *P.aeruginosa DR4877/07* strain showed remarkable resistance to Gentamycin that we could not determine the MIC. Therefore, FICI was determined against the *P. aeruginosa ATCC 9027* strains.

Table S2. MIC of B2088 and other antibiotics against 3 different strains of *K. pneuminae*

Strains	MIC (μg/mL) of				
	**B2088**	**Tobramycin**	**Gentamycin**	**Gatifloxacin**	**Chloramphenicol**
K. pneumoniae 4299	12.5	0.024	0.39	1.56	12.5
K. pneumoniae 07955	6.25	25	100	1.56	50
K. pneumoniae 31158	12.5	0.012	0.05	1.56	3.125
